# Nomogram predicting overall survival of stage IIIB non‐small‐cell lung cancer patients based on the SEER database

**DOI:** 10.1111/crj.13660

**Published:** 2023-07-19

**Authors:** Ziye Li, Pingfan Shi, Chenge Qin, Wen Zhang, Shumeng Lin, Tiansheng Zheng, Ming Li, Lihong Fan

**Affiliations:** ^1^ Integrated Chinese and Western Medicine Pulmonary Nodules Center, Shanghai Tenth People's Hospital, School of Medicine Tongji University Shanghai China; ^2^ Department of Respiratory Medicine, Shanghai Tenth People's Hospital Tongji University School of Medicine Shanghai China; ^3^ Medical School of Nantong University Nantong University Nantong China

**Keywords:** nomogram, non‐small‐cell lung cancer, overall survival, prognosis, stage IIIB, the Surveillance, Epidemiology, and End Results (SEER) database

## Abstract

**Purpose:**

We aimed to evaluate the prognostic value of stage IIIB non‐small‐cell (NSCLC) lung cancer patients and to construct a nomogram to effectively predict their overall survival (OS).

**Methods:**

In total, 4323 patients with stage IIIB NSCLC diagnosed between 1975 and 2018 were selected from the Surveillance, Epidemiology, and End Results (SEER) database. Multiple prognostic factors were combined to construct a nomogram for predicting OS of patients with stage IIIB NSCLC. The discrimination and calibration of the nomogram were evaluated by C‐indexes and calibration curves. The nomogram was evaluated for predictive ability using receiver operating characteristic (ROC) curves, decision curve analysis curve (DCA), and clinical impact curve (CIC).

**Results:**

The nomogram was built on data of 4323 patients with stage IIIB NSCLC and consisted of the following prognostic factors: age, race, sex, primary labeled, pathology, T stage, whether to receive surgery, whether to receive radiotherapy, and whether to receive chemotherapy. The C‐index in the training and validation sets for the nomogram was 0.672 (95% CI: 0.661–0.683) and 0.675 (95% CI: 0.656–0.694), respectively. According to scores of the nomogram, patients in the complete set, validation set, and training set were classified into two risk groups, low risk and high risk.

**Conclusions:**

We developed the first validated nomogram to estimate the OS of patients with stage IIIB NSCLC. The nomogram was based on nine prognostic factors and provided an individualized risk estimate of 3‐year and 5‐year OS survival in patients with stage IIIB NSCLC.

## Graphical abstract

The nomogram was built on data of 4323 patients with stage IIIB NSCLC and consisted of the following prognostic factors: age, race, sex, primary labeled, pathology, T stage, whether to receive surgery, whether to receive radiotherapy, and whether to receive chemotherapy, and the model demonstrated excellent predictive performance.

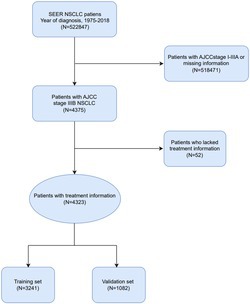

## 1 INTRODUCTION

Lung cancer is one of the leading causes of cancer‐related deaths worldwide.^1^ According to the classification of lung cancer morphology, it can be divided into small cell lung cancer (SCLC) and non‐small cell lung cancer (NSCLC), of which NSCLC accounts for about 80%–85%^2^, ^3^ of all lung cancers. More than 60% of lung cancer patients have locally advanced or metastatic disease (stage III or IV) at diagnosis,^3^, ^4^ and the 5‐year survival rate of IIIB NSCLC patients was 26%.^5^ Therefore, accurate assessment of survival prognosis of patients with stage IIIB NSCLC is of great significance for clinical guidance.

Traditionally, the American Joint Committee on Cancer Tumor Node Metastasis (AJCC TNM) staging system was widely used in the prognostic assessment of NSCLC patients and provides important guidance on patient tumor staging and prognosis.^6^ However, it does not enable accurate assessment of prognosis, according to AJCC stratification based on tumor size and invasion, as well as lymph node involvement.^7^ Other independent prognostic factors, such as sex, age, stage,^8^, ^9^ and treatment‐related factors,^7^ could greatly contribute to individualized survival prediction. Therefore, there is an urgent need to develop a comprehensive and individualized assessment system for patients with stage IIIB NSCLC.

Nomogram can include multiple prognostic risk factors to provide an individualized assessment of clinical patients by combining important pathological and clinical hallmarks of the tumor.^10^, ^11^ At the same time, it also provides a more intuitive reference for clinical decision making.^12^ Currently, there is no nomogram used to predict the survival prognosis of patients with stage IIIB NSCLC.

This study included almost all patients with stage IIIB non‐small cell lung cancer from the Surveillance, Epidemiology and End Results (SEER) database to analyze the clinical characteristics and treatment options. We developed an intuitive and simple nomogram of clinical prognosis for patients with stage IIIB NSCLC, accurately predict their survival prognosis, and provide an easy‐to‐use and effective reference for clinical decision making.

## 2 METHODS

### 2.1 Data sources

Using the SEER database of the National Cancer Institute (NCI), we collected data from January 1, 1975, to December 31, 2018. As a population‐based cancer registry, the SEER program was launched in 1973 in the United States and covers approximately 28% of the US population. The patient sample for this study was selected from publicly de‐identified data from the NCI SEER 18 registry, which is allowed to be used in relevant medical research and is considered exempt from institutional review board oversight. In addition, the annual follow‐up rate for all patients diagnosed with cancer in the past 5 years was 90%.

### 2.2 Patient choice

Inclusion criterion for this retrospective study was patients with NSCLC with stage IIIB disease according to the joint council on cancer (AJCC) edition 7. Exclusion criterion was patients lacking survival information. Complete patient information is obtained from the SEER database.

### 2.3 Construction and validation of the prognostic model

According to the requirements of data analysis, patients with incomplete data on clinical characteristics (including gender, age, survival time, and survival status) were excluded from the construction of this model. Using the random number table method, the samples with complete survival information were randomly divided into training set and validation set according to the ratio of 3:1. The chi‐square test was used to detect whether there was a difference in clinical information between the training set and the validation set. Subsequently, univariate logistic regression analysis was performed on the training set to screen clinical features associated with prognosis. Multivariate logistic regression analysis was performed for clinical features with *P* < 0.05, and a model for predicting risk was constructed. All tumor patients were divided into high‐risk and low‐risk groups according to the median risk value. Survival curves were drawn using the Kaplan–Meier method and log‐rank test. A nomogram was constructed using the R rms package, and a calibration curve was drawn to evaluate the predictive performance of the nomogram. The receiver operating characteristic (ROC) was plotted to calculate the area under the curve (AUC) predicted by the nomogram model to evaluate the performance of the model. At the same time, we also calculated whether the patients received the guideline‐recommended treatment modality chemotherapy to predict survival and prognosis efficacy. In addition, we constructed the decision curve analysis (DCA) and the clinical impact curve (CIC) to further validate the performance of the nomogram prediction model.

### 2.4 Statistical analysis

The chi‐square test was used to evaluate the differences in the distribution of various clinical data in the training and validation sets. Kaplan–Meier analysis and log‐rank test assessed differences in survival between high‐ and low‐risk groups. Independent prognostic factors for OS were determined by univariate and multivariate logistic regression analysis. All statistical analyses were performed using R software (Version 4.0.2). *P* < 0.05 means the difference is statistically significant.

## 3 RESULTS

### 3.1 Clinical characteristics of patients

A total of 522 847 NSCLC patients were collected from the SEER database. After excluding 518 471 NSCLC patients without stage information or with AJCC7 stage I–IIIA and IV, 4375 NSCLC patients with stage IIIB disease were screened out, including 52 NSCLC patients with missing treatment information. A total of 4323 eligible stage IIIB NSCLC patients were included in this study, which were divided into training set and validation set by random number table method, including 3241 training set and 1082 validation set. There was no difference in clinical characteristics between the training set and validation set (All *P* > 0.05). The basic information of the patients is shown in Table 1.

**TABLE 1:** Demographic and clinical characteristics of patients with stage IIIB NSCLC.^a^

Characteristics	No.(%)			
	Training set	Validation set	*P*	SD
Race (*n*, %)			0.604	0.22
Caucasians	2498(77.1)	812(75.0)		
African Americans	404(12.5)	162(15.0)		
Others	336(10.3)	107(9.9)		
Unknown	3(0.1)	1(0.1)		
Sex (*n*, %)			0.423	0.17
Male	1793(55.3)	579(53.5)		
Female	1448(44.7)	503(46.5)		
Age of diagnosis			0.179	0.22
20–39	16(0.5)	5(0.5)		
40–59	711(21.9)	223(20.6)		
60–79	2013(62.1)	660(61.0)		
≥80	501(15.5)	194(17.9)		
Primary labeled			0.663	0.4
Main bronchus	210(6.5)	65(6.0)		
Upper lobe	1863(57.5)	613(56.7)		
Middle lobe	128(3.9)	52(4.8)		
Lower lobe	671(20.7)	224(20.7)		
Overlapping	26(0.8)	12(1.1)		
Nos	343(10.6)	116(10.7)		
Laterality			0.449	0.18
Right	1265(39.0)	404(37.3)		
Left	1879(58.0)	644(59.5)		
Other	97(3.0)	34(3.2)		
Pathology			0.776	0.26
Large cell carcinoma	64(2.0)	14(1.3)		
Squamous cell carcinoma	1221(37.7)	438(40.5)		
Adenocarcinoma	1378(42.5)	432(39.9)		
Others	578(17.8)	198(18.3)		
T stage			0.774	0.39
T0	24(0.7)	7(0.6)		
T1	308(9.5)	106(9.8)		
T2	569(17.6)	180(16.6)		
T3	392(12.2)	132(12.2)		
T4	1800(55.5)	604(55.8)		
TX	146(4.5)	53(5.0)		
N stage			0.922	0.17
N2	1403(43.3)	466(43.1)		
N3	1838(56.7)	616(56.9)		
Surgery			0.266	0.07
No	3053(94.2)	1031(95.3)		
Yes	188(5.8)	51(4.7)		
Radiotherapy			0.946	0.16
No	1080(33.3)	359(33.2)		
Yes	2161(66.7)	723(66.8)		
Chemotherapy			0.373	0.16
No	970(29.9)	344(31.8)		
Yes	2271(70.1)	738(68.2)		
Marital status			0.526	0.60
Single	474(14.6)	155(14.3)		
Married	1612(49.7)	556(51.4)		
Divorced	448(13.8)	148(13.7)		
Separated	35(1.1)	12(1.1)		
Domestic partner	11(0.3)	2(0.2)		
Widowed	525(16.3)	169(15.6)		
Unknown	136(4.2)	40(3.7)		

^a^ For categorical variables, *P* values were analyzed by chi‐square tests.

**FIGURE 1:**
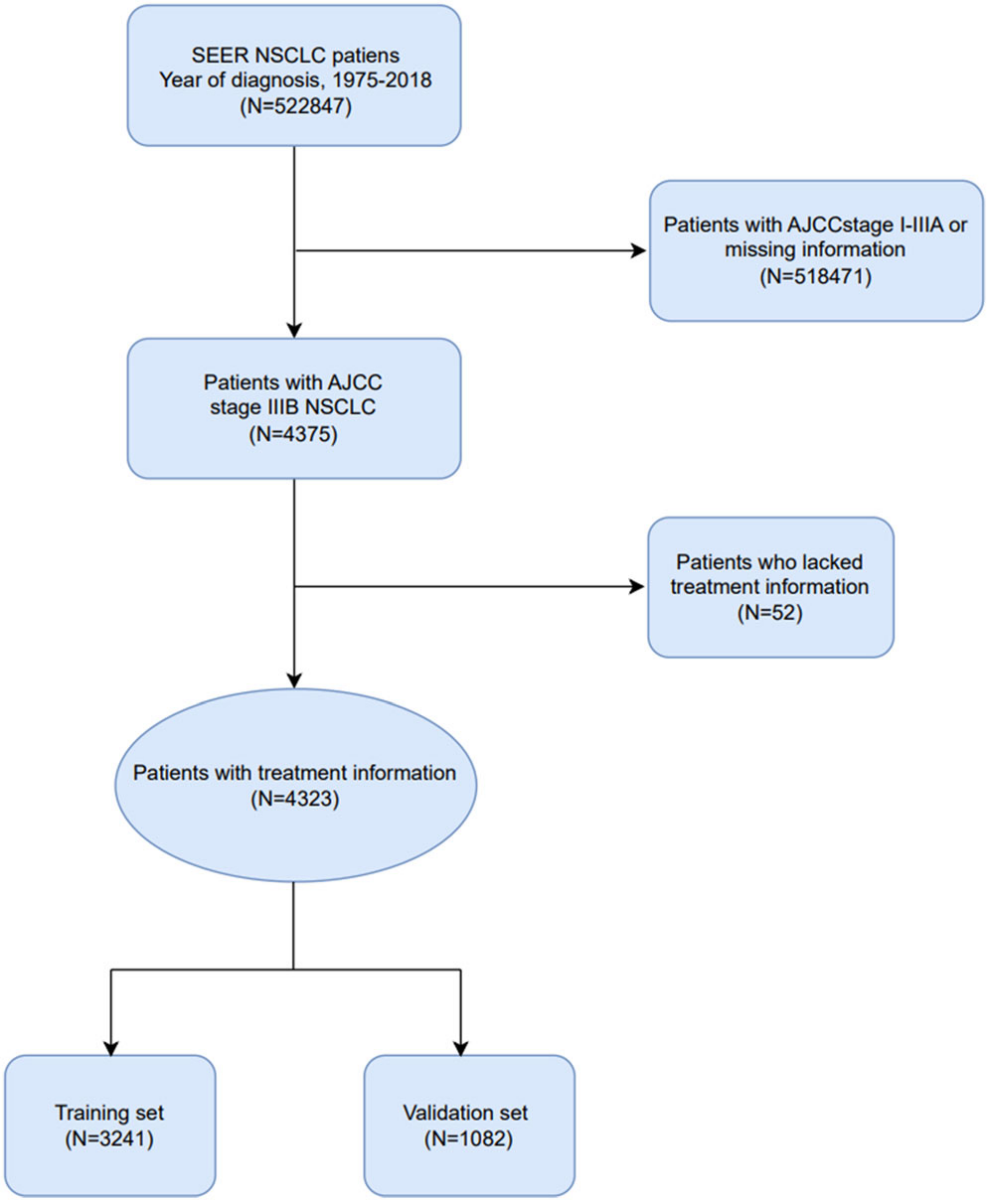
Flowchart of patient selection steps.

Among them, 21 (0.5%) were 20–39 years old, 934 (21.6%) were 40–59 years old, 2673 (61.8%) were 60–79 years old, and 695 (16.1%) were ≥80 years old. There were slightly more males (2372/54.9%) than females (1951/45.1%), more Caucasians (3310/76.6%) than African Americans (566/13.1%), other cases (443/10.2%), and unknown cases (4/0.1%). Among the pathological types, squamous carcinoma (1659/38.4%) and adenocarcinoma (1810/41.9%) accounted for the majority, large cell carcinoma (78 cases) accounted for 1.8%, and other pathological types (776 cases) accounted for 17.9%. Most of the patients (4084/94.5%) did not receive surgical treatment, 3009 (69.6%) received chemotherapy, and 2884 (66.7%) received radiotherapy. In the study, all patients were diagnosed between 1975 and 2018 (Figure 1).

### 3.2 Analysis of prognostic factors in stage IIIB NSCLC patients

The univariate and multivariate analysis results of prognostic factors in the training set are shown in Table 2. Univariate analysis showed that age, race, sex, primary labeled, pathology, T stage, whether to receive surgery, whether to receive radiotherapy, whether to receive chemotherapy, and marital status were all related factors affecting the prognosis of patients (*P* < 0.05). Incorporating these factors into a multivariate analysis, the results showed that age, race, sex, primary labeled, pathology, T stage, whether to receive surgery, whether to receive radiotherapy, and whether to receive chemotherapy were the related factors affecting the prognosis of the patients (*P* < 0.05).

**TABLE 2:** Univariate and multivariate analysis results.^a^

Characteristics	Univariate analysis		Multivariate analysis	
	OR (95% CI)	*P*	OR (95% CI)	*P*
Age		<0.001		<0.001
20–39	1		1	
40–59	2.475(1.011–6.058)	0.047	2.349(0.880–6.272)	0.088
60–79	3.891(1.602–9.453)	0.003	3.009(1.132–8.000)	0.027
80+	11.604(4.515–29.828)	<0.001	5.288(1.871–14.945)	0.002
Race		0.029		0.014
Caucasians	1		1	
African Americans	0.978(0.753–1.271)	0.868	1.056(0.797–1.398)	0.704
Other	0.678(0.522–0.880)	0.004	0.643(0.487–0,848)	0.002
Unknown	0.455(0.047–4.385)	0.496	0.628(0.034–11.712)	0.755
Sex		0.008		0.005
Female	1		1	
Male	1.264(1.063–1.504)	0.008	1.310(1.084–1.584)	0.005
Laterality		0.925		
Right	1			
Left	1.028(0.859–1.229)	0.764		
Other	1.088(0.642–1.845)	0.754		
Labeled		<0.001		0.001
Main bronchus	1		1	
Upper lobe	0.448(0.283–0.708)	0.001	0.451(0.280–0.727)	0.001
Middle lobe	0.430(0.237–0.782)	0.006	0.466(0.249–0.873)	0.017
Lower lobe	0.672(0.411–1.100)	0.114	0.657(0.393–1.100)	0.110
Overlapping	0.965(0.274–3.401)	0.955	1.221(0.291–5.114)	0.785
NOS	0.583(0.345–0.985)	0.044	0.600(0.342–1.054)	0.076
Pathology		<0.001		0.007
Large cell	1		1	
Squamous cell	1.654(0.894–3.060)	0.109	1.443(0.760–2.737)	0.262
Adenocarcinoma	0.876(0.519–1.750)	0.876	1.007(0.535–1.896)	0.983
Other	1.564–2.952	0.168	1.294(0.668–2.508)	0.445
T stage		0.013		0.008
T0	1		1	
T1	1.849(0.819–4.177)	0.139	1.972(0.801–4.859)	0.140
T2	2.509(1.125–5.598)	0.025	2.664(1.098–6.464)	0.030
T3	2.953(1.303–6.694)	0.010	3.339(1.356–8.225)	0.009
T4	2.733(1.247–5.989)	0.012	2.844(1.202–6.727)	0.017
TX	2.498(1.044–5.978)	0.040	1.874(0.747–4.704)	0.181
N stage		0.802		
N2	1			
N3	0.978(0.821–1.165)	0.802		
Surgery		<0.001		<0.001
No	1		1	
Yes	0.274(0.207–0.364)	<0.001	0.274(0.201–0.372)	<0.001
Radiotherapy		<0.001		<0.001
No	1		1	
Yes	0.376(0.301–0.469)	<0.001	0.542(0.423–0.694)	<0.001
Chemotherapy		<0.001		<0.001
No	1		1	
Yes	0.213(0.161–0.282)	<0.001	0.350(0.258–0.474)	<0.001
Marital		<0.001		0.062
Single	1		1	
Married	0.849(0.657–1.095)	0.207	0.874(0.642–1.117)	0.239
Divorced	1.190(0.849–1.668)	0.312	1.189(0.832–1.699)	0.342
Separated	0.586(0.281–1.222)	0.154	0.643(0.299–1.382)	0.258
Domestic partner	0.528(0.142–1.957)	0.339	0.517(0.129–2.074)	0.352
Widowed	1.705(1.201–2.420)	0.033	1.272(0.867–1.867)	0.218
Unknown	0.957(0.592–1.547)	0.856	0.898(0.541–1.491)	0.678

^a^ For categorical variables, *P* values were analyzed by chi‐square tests.

### 3.3 Nomogram construction and evaluation

**FIGURE 2:**
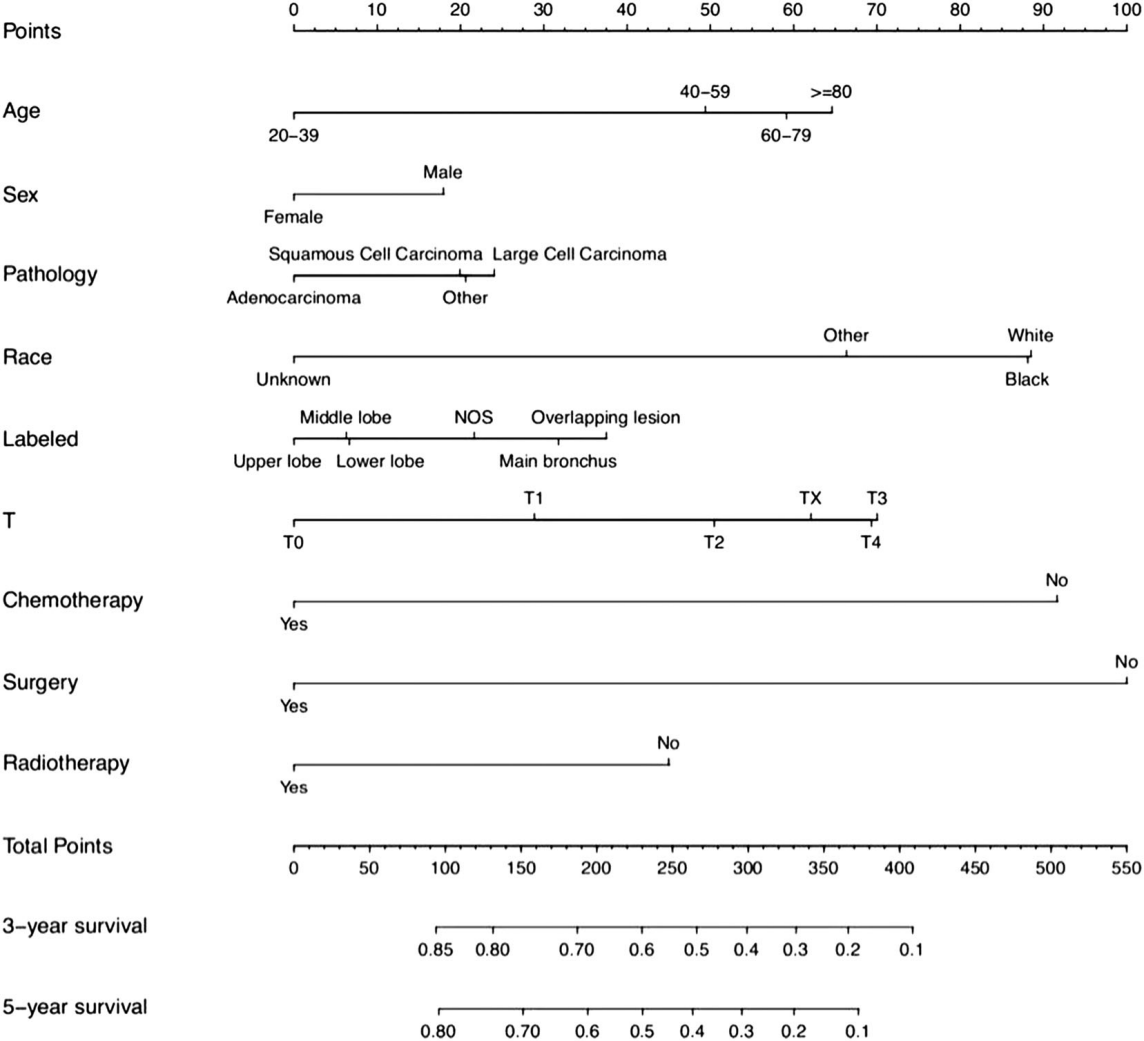
Prediction in patients with stage IIIB NSCLC nomogram for 3‐year and 5‐year OS survival.

**FIGURE 3:**
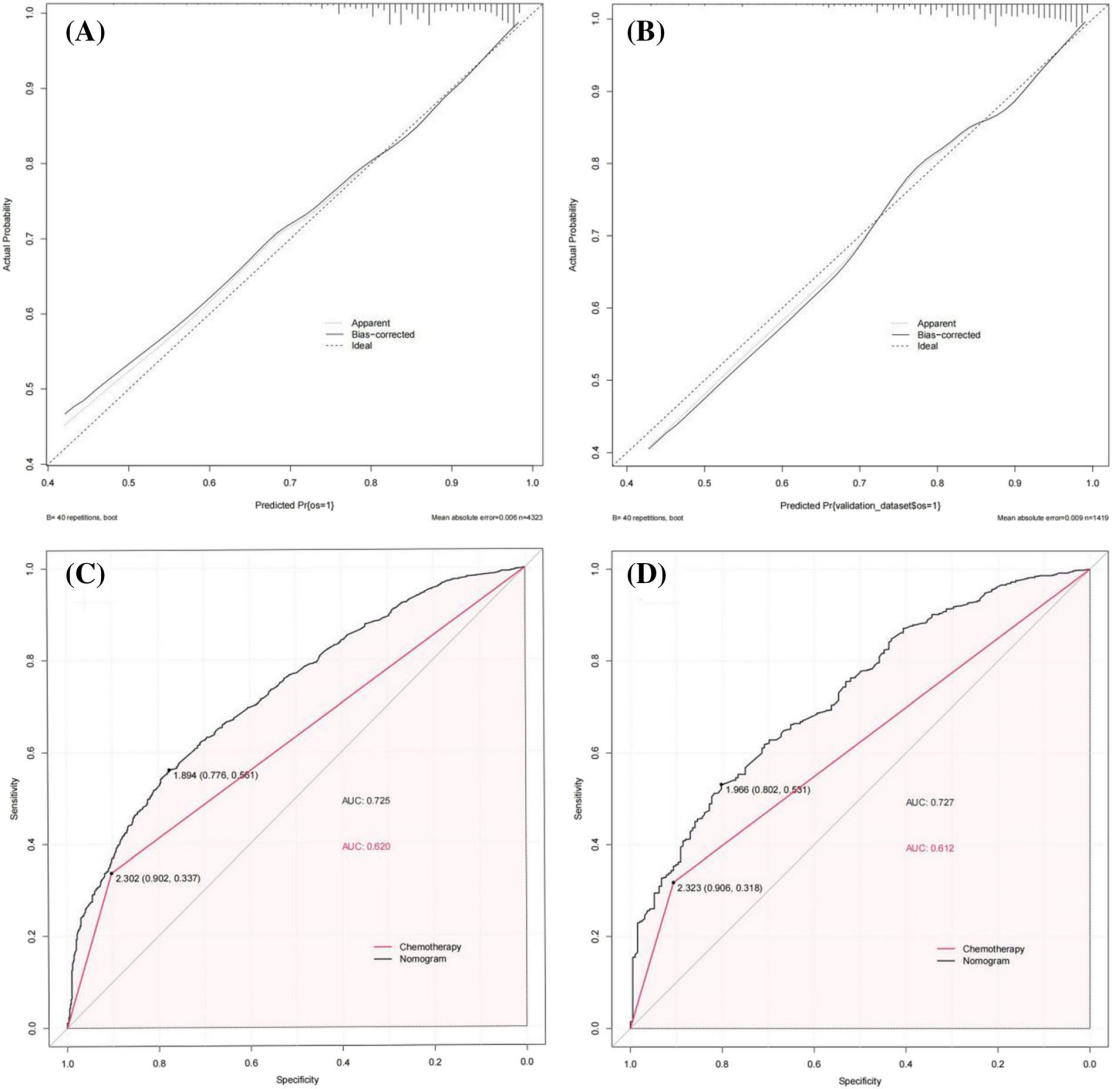
Patients with stage IIIB NSCLC nomogram of calibration and ROC curve analysis results. (A) Calibration curve analysis of the line graph in the training set. (B) Calibration curve analysis of the line graph in the validation set. Ideal stands for perfect prediction. Apparent values indicate obvious estimates of predicted and observed values; at the same time, bias‐corrected (which stands for bias) displays the corrected estimates by using 1000 autonomous samples. (C) The ROC curve of the overall survival rate of the rosettes in the training set, and the area under the curve was 0.725. (D) The ROC curve of the overall survival rate of the rosettes in the validation set, and the area under the curve was 0.727.

**FIGURE 4:**
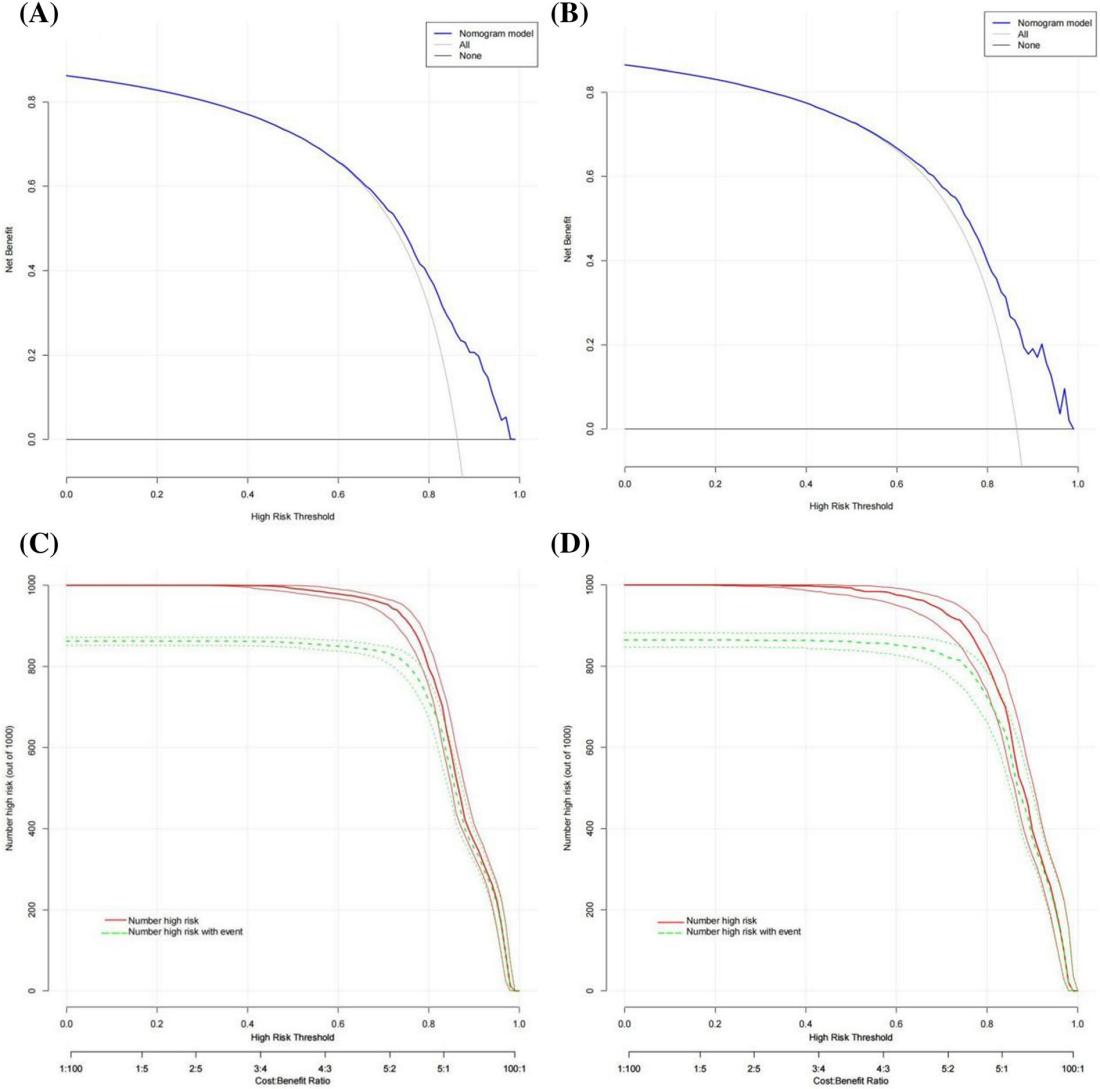
Patients with stage IIIB NSCLC nomogram DCA curves with CIC curve results. (A) DCA curve of training set. (B) DCA curve of validation set. When a patient's risk threshold probability of death is between 0.6 and 1.0, a greater net benefit can be obtained when clinical decisions are made using a rosette than with “no treatment” or “all treatment” options. (C) CIC curve of training set. (D) CIC curve of validation set. The solid red curve (number of high‐risk individuals) represents the number of people classified as positive (high risk) by the prediction model at each threshold probability; the dashed green curve (number of high‐risk individuals with outcomes) is the true number of positives at each threshold probability.

Nomogram for predicting 3‐year and 5‐year OS survival in stage IIIB patients was constructed using the above independent risk factors, as shown in Figure 2. Nomogram showed that surgery had the greatest impact on prognosis, followed by chemotherapy, race, T stage, age, radiotherapy, primary labeled, pathology, and sex. By adding the scores for each of the above variables, a patient's individual probability of survival can be calculated. The C‐index training set and validation set of nomogram were 0.672 (95% CI: 0.661–0.683) and 0.675 (95% CI: 0.6566–0.694), respectively. The calibration curve showed that the prediction fitted well with the actual observation (slope of training set was 0.903, intercept was 0.006; the slope of the validation set was 0.901, and the intercept was 0.011), indicating that the model had high prediction efficiency (Figure 3A,B). ROC curves of the overall survival rates of stage IIIB NSCLC patients in the training and validation set were plotted (Figure 3C,D), whose AUCs were 0.725 and 0.727, respectively. Nomogram had a higher AUC and showed better predictive efficacy without significant overfitting in both the training set and the validation set compared with chemotherapy as recommended by the guidelines (*P* < 0.001). To further evaluate the clinical value of the nomogram, the DCA curve for overall survival was drawn (Figure 4A,B), which showed that when a patient's risk threshold probability of death was between 0.6 and 1.0, a greater net benefit could be obtained when using the rosette for clinical decision making than “no treatment” or “all treatment” options. The CIC analysis showed that the model was effective. When the threshold probability was greater than 80% of the predicted score probability value, the prediction model determined that the patients with high risk of death of stage IIIB NSCLC were highly matched with the actual patients with stage IIIB NSCLC death, confirming the high clinical efficiency of the prediction model (Figure 4C,D).

### 3.4 Survival analysis

**FIGURE 5:**
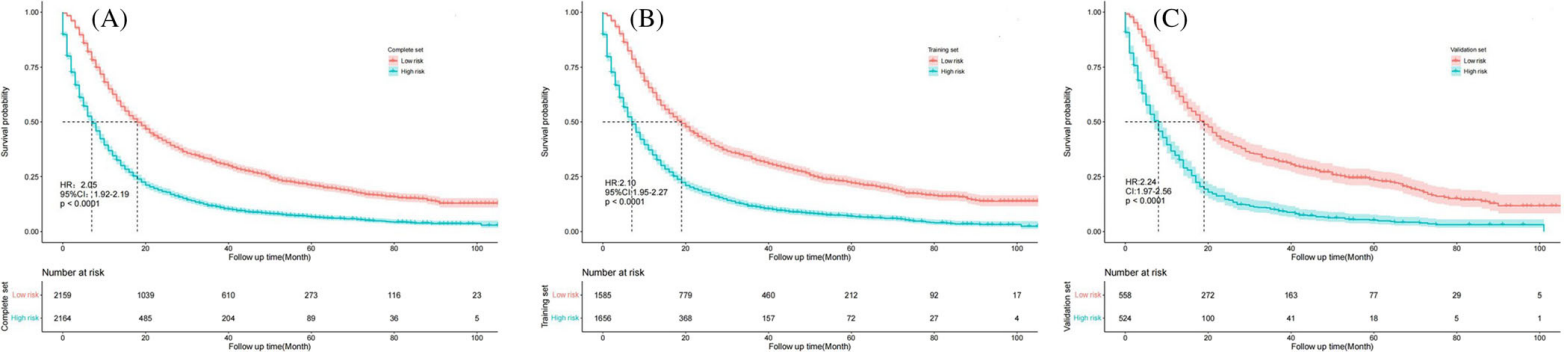
Kaplan–Meier survival curve of patients with high and low risk. (A) Complete set. (B) Training set. (C) Validation set.

After calculating the nomogram score of stage IIIB NSCLC patients in the training set, the optimal risk score of 215.79 was obtained, which was used to divide the patients in the complete set, training set, and validation set into high‐ and low‐risk groups. In the complete set, median overall survival was 18 months for low‐risk patients and 7 months for high‐risk patients (HR: 2.05, 95% CI: 1.92–2.19, *P* < 0.001). The survival curves were shown in Figure 5A. In the training set, median overall survival was 19 months for low‐risk patients and 7 months for high‐risk patients (HR: 2.10, 95% CI: 1.95–2.27, *P* < 0.001), and survival curve was shown in Figure 5B. In the validation set, the median overall survival was 17 months for low‐risk patients and 7 months for high‐risk patients (HR: 2.24, 95% CI: 1.97–2.56, *P* < 0.001), and the survival curve was shown in Figure 5C. Therefore, the use of this line graph model can accurately predict the survival prognosis of stage IIIB NSCLC patients.

## 4 DISCUSSION

In this study, we used the SEER database to develop the first nomogram to predict the prognosis of patients with stage IIIB NSCLC. A total of 4323 patients with stage IIIB NSCLC and nine risk factors were included. By ROC analysis, the predictive model was confirmed to have high clinical validity. Validation by DCA and CIC confirmed that the nomogram can accurately predict survival of stage IIIB NSCLC patients. Based on the scores of the nomogram, patients with stage IIIB NSCLC were classified into two risk groups, high and low, with the training set overall survival of 19 and 7 months, respectively.

Traditionally, the TNM staging system was used for prognostic assessment of patients with advanced NSCLC.^13^ However, the TNM staging system considered patients with stage IIIB NSCLC as a whole, ignoring the impact of patients' clinical characteristics and independent risk factors on prognosis. Thus, the prognostic assessment of patients with stage IIIB NSCLC was lower in sensitivity and specificity, and the assessment efficacy was poorer. Sun et al established and validated a nomogram for predicting lung cancer‐specific survival (LCSS) and OS in elderly patients with metastatic NSCLC. They find that patients with poor prognosis were mainly concentrated in patients with a diagnosis age of ≥80 years^14^; our research also supported this conclusion. Despite previous reports of prognostic assessment models for NSCLC patients,^15^, ^16^, ^17^, ^18^ there is no nomogram for prognostic assessment of patients with stage IIIB NSCLC. This nomogram was built based on multiple risk factors, including age, race, sex, primary labeled, pathology, T‐stage, whether to receive surgery, whether to receive radiotherapy, and whether to receive chemotherapy, to individually evaluate patients with stage IIIB NSCLC. According to our results, the C‐indexes of the sets for training and validating the nomogram were 0.672 (95% CI: 0.661–0.683) and 0.675 (95% CI: 0.6566–0.694), which is higher than the TNM staging (0.582, 95% CI: 0.545–0.619, *P* < 0.01).^19^ Therefore, the nomogram is a reliable tool to predict the prognosis of patients with stage IIIB NSCLC.

Furthermore, the calibration curve showed that the prediction fitted well with the actual observation, indicating that the model had high prediction efficiency. By ROC curve analysis, the AUCs of the training set and validation set were 0.725 and 0.727, showing better prediction performance. DCA determines the validity of nomogram for predicting overall survival. CIC analysis showed high clinical validity of this model. Our results suggest that the nomogram can comprehensively and accurately predict the prognosis of stage IIIB NSCLC patients, which has significant implications for clinical guidance.

Our study has the following advantages. First, we included a large number of patients, 4323 patients with stage IIIB NSCLC from the SEER database. Second, the nomogram comprehensively contained multiple factors affecting the patient's prognosis, and the sensitivity and specificity of the prognostic assessment was high. Third, the accuracy of the prediction of the nomogram was validated by various ways, and the efficacy of the assessment of clinical prognosis was relatively significant. Fourth, the nomogram was shared on the web (https://spfcy.shinyapps.io/IIIBNSCLCNomapp/) for easy clinical use and promotion to assist in clinical decision making. However, this study still has some limitations. It was retrospective, which leads to unavoidable selection bias. Finally, the SEER database lacks clinical information of patients and performance status (PS), which may also affect the accuracy of the prediction of this model.

## 5 CONCLUSION

We developed the validated nomogram to estimate the OS of patients with stage IIIB NSCLC. The nomogram was based on age, race, sex, primary labeled, pathology, T stage, whether to receive surgery, whether to receive radiotherapy, and whether to receive chemotherapy and provided an individualized risk estimate of 3‐year and 5‐year OS survival in patients with stage IIIB NSCLC. The prediction results have a relatively good performance.

## AUTHOR CONTRIBUTIONS

Ziye Li, Pingfan Shi, Ming Li, Wen Zhang, Shumeng Lin, Tiansheng Zheng, and Lihong Fan contributed to conception and design of the study. Ziye Li and Pingfan Shi performed the data extraction and statistical analysis. Ziye Li and Pingfan Shi wrote the main manuscripts. All authors reviewed and approved the final version of the manuscript. All authors reviewed and approved the final version of the manuscript.

## CONFLICT INTEREST STATEMENT

The authors declare that they have no competing interests.

## ETHICS STATEMENT

Patients from the Surveillance, Epidemiology, and End Results (SEER) database had previously consented to participate in any scientific research worldwide. Informed consent was obtained from all individual participants included in the study. All authors approved the manuscript to publish.

## Data Availability

Publicly available datasets were analyzed in this study. These data can be found here: https://seer.cancer.gov/data/. The nomogram can be founded on the web: https://spfcy.shinyapps.io/IIIBNSCLCNomapp/.
